# Intact parathyroid hormone and whole parathyroid hormone assay results disagree in hemodialysis patients under cinacalcet hydrochloride therapy

**DOI:** 10.1007/s10157-014-1045-3

**Published:** 2014-11-11

**Authors:** Ryo Koda, Junichiro James Kazama, Koji Matsuo, Kazuko Kawamura, Suguru Yamamoto, Minako Wakasugi, Tetsuro Takeda, Ichiei Narita

**Affiliations:** 1 Department of Clinical Nephrology and Rheumatology, Niigata University Graduate School of Medical and Dental Sciences, Niigata, Japan; 2 Department of Nephrology, Dokkyo University Koshigaya Medical Center, Koshigaya, Japan; 3 Division of Blood Purification Therapy, Niigata University Medical and Dental Hospital, 1-754 Asahimachi-Diri, Chuo-ku, Niigata, Niigata 951-8510 Japan; 4 Center of Interorgan Research, Niigata University Graduate School of Medical and Dental Hospital, Niigata, Japan

**Keywords:** Intact PTH, Whole PTH, Cinacalcet hydrochloride, Hemodialysis

## Abstract

**Background:**

The parathyroid gland secretes 1-84 and 7-84 parathyroid hormone (PTH) fragments, and its regulation is dependent on stimulation of the extracellular calcium-sensing receptor. While the intact PTH system detects both PTH fragments, the whole PTH system detects the 1-84PTH but not the 7-84PTH. Cinacalcet hydrochloride (CH) binds to calcium-sensing receptor as a calcimimetic. Here we investigated the role of CH treatment in the assessment of parathyroid gland function.

**Methods:**

Stable adult dialysis patients for whom CH therapy was planned were included. Patients for whom CH therapy was not planned were simultaneously included as the control group.

**Results:**

The CH group (*n* = 44) showed significantly higher circulating levels of Ca, intact PTH, and whole PTH, before the CH treatment than the control group (*n* = 112). The Ca, intact PTH, and whole PTH levels decreased along with the CH therapy, and the Ca levels became comparable in the 8th week of treatment and thereafter. The CH group in the 8th week and thereafter showed significantly lower whole/intact PTH ratios than the control group, while the whole/intact PTH ratio was not significantly different between before and during the CH therapy. A multiple regression analysis revealed that the whole/intact PTH ratio was almost constant, but both the serum Ca level and a CH therapy could potentially modify the fixed number. When the whole PTH levels were estimated by intact PTH levels using the relationship between them in the control group, the levels were clearly overestimated in the CH group.

**Conclusions:**

Although the direct effect of CH on the whole/intact PTH ratio is masked by its hypocalcemic action, we could successfully demonstrate that the ratio in CH users is lower than that in the non-users with comparable levels of serum Ca. Evaluating parathyroid function with intact PTH according to the clinical practice guidelines in patients being treated with CH may lead to significant overestimation and subsequent overtreatment.

## Introduction

The secretion of parathyroid hormone (PTH) by parathyroid cells is controlled by extracellular calcium (Ca) levels [1]. The sensor system that transmits the information of extracellular Ca levels into the nuclei of parathyroid cells involves the Ca-sensing receptor [2]. Cinacalcet hydrochloride (CH) also binds to the Ca-sensing receptor, and it transmits false information of elevated extracellular Ca levels into the cells [3]. As a result, CH suppresses the PTH secretion and production in parathyroid cells. CH is thus currently used for the treatment of secondary hyperparathyroidism in chronic kidney disease (CKD) patients with satisfactory clinical effects [4, 5].

In addition to 1-84PTH, which actually induces hormonal action within the target cells, various PTH fragments of other lengths are also found in the systemic circulation. Among these fragments, 7-84PTH is directly secreted from parathyroid cells, and binds to PTH-I receptors with a binding affinity comparable to that of 1-84PTH [6]. The extracellular Ca level critically affects the switching of 1-84PTH/7-84PTH secretion in parathyroid cells. That is, 7-84PTH is predominantly secreted from parathyroid cells under a hypercalcemic condition, whereas 1-84PTH secretion is promoted in these cells under a hypocalcemic condition [7, 8]. Since total PTH levels are also increased under a hypocalcemic condition, the change in the serum 1-84PTH level is greatly enhanced by the extracellular Ca level. Because the extracellular Ca levels are sensed through the Ca-sensing receptor, it has been hypothesized that in patients being treated with CH, the secretion of 1-84PTH is suppressed, while 7-84PTH secretion is promoted.

The so-called intact PTH assay detects both 1-84PTH and 7-84PTH fragments. In contrast, the 3rd generation PTH assay, also known as the whole PTH assay, does not detect 7-84PTH fragments because one of the epitopes for the sandwich immunoassay is located at the very N-terminal of the PTH molecule [9]. Strictly speaking, the whole PTH assay does detect some fragments other than true 1-84 PTH [10]. However, the majority of investigators would agree that the whole PTH assay detects functioning PTH molecules more specifically than the intact PTH assay does.

The levels of intact PTH and whole PTH do show a tight correlation [11]. However, the whole/intact PTH ratio, which is practically indicating the ratio of 1-84PTH and the sum of 1-84PTH + 7-84PTH, varies widely among individuals, and some clinical investigators insist that the ratio is an indicator of bone turnover [12]. In addition, vitamin D receptor activator treatment has been shown to slightly decrease the whole/intact PTH ratio in dialysis patients with secondary hyperparathyroidism, presumably through its calcemic action [13]. Since, CH therapy directly suppresses 1-84PTH secretion while promoting 7-84PTH secretion, in theory, its effect on the whole/intact PTH ratio change might be much greater. If CH therapy has a major effect on the whole/intact PTH ratio, then it would no longer be possible to estimate 1-84PTH levels from intact PTH levels.

To address these issues, we performed a prospective clinical study. The aim of this study was to validate the efficacy of PTH assays for evaluating parathyroid function in dialysis patients under CH therapy.

## Patients and methods

### Patients

Adult CKD patients who had been under stable maintenance hemodialysis therapy for more than 1 year at Niigata University Medical and Dental Hospital or one of its related facilities were enrolled. All subjects were dialysis patients for whom CH therapy was considered due to prolonged hyperactivation of parathyroid function despite of other medical therapy in accordance with the Clinical Practice Guidelines for CKD–MBD issued by the Japanese Society for Dialysis Therapy (JSDT guidelines) [14]. The exclusion criteria were as follows: (1) hypocalcemia defined by serum Ca levels <9.0 mg/dL; (2) the presence of any contraindications for CH therapy; (3) past history of kidney transplantation or parathyroid intervention therapies; and (4) complications of malignant tumor, severe cardiac failure, chronic inflammatory disease, or any other diseases that could disturb stable hemodialysis therapy. All the enrolled patients provided written informed consent to participate in the study. With respect to the registration of CH patients, the simultaneous registration of one or more control patients with mild secondary hyperparathyroidism, who had not received and would not need CH therapy, was recommended. However, the registration of control patients was not compulsory if no applicable patients were available.

### Methods

All the patients’ blood samples were collected at 9:00 a.m. after a 12-h period of fasting. The initial dosage of CH was 25 mg/day. In accordance with the JSDT guidelines, the dosage of CH was thereafter freely changed in order to maintain the intact PTH levels in the patients in the CH group. However, the minimal dosage was set at 12.5 mg/day, and those patients who required a decrease in the dosage of CH to <12.5 mg were excluded from the analyses. No limitation was set for using vitamin D receptor activators, phosphate binders, antihypertensive agents, or erythropoietin receptor stimulating agents.

**Fig. 1:**
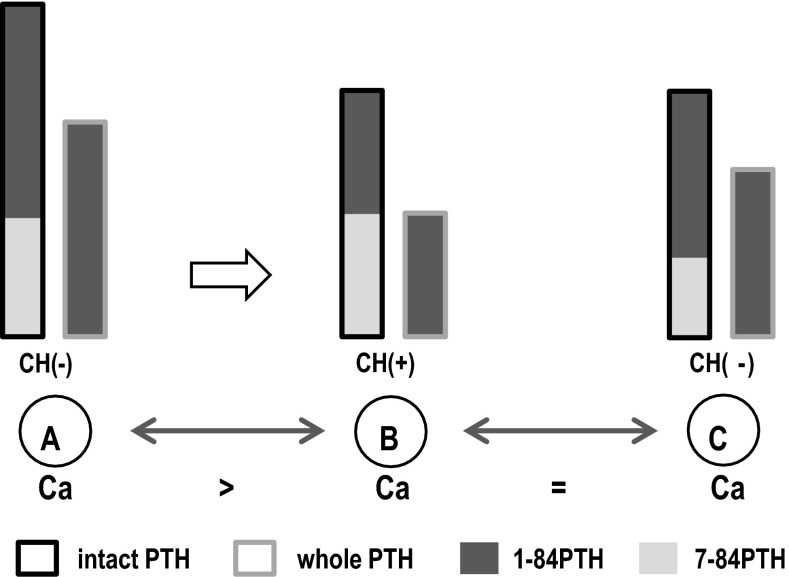
Generally, the serum Ca concentration decreased along with the time course of CH therapy. Since the serum Ca level is another important determinant of the whole/intact PTH ratio, the pure effect of CH on the whole/intact PTH ratio cannot be evaluated by comparing the data obtained from the same patients between before (*A*) and during/after (*B*) CH therapy. Therefore, we established a control group of patients (*C*) whose parathyroid functions were stable even without using CH in this protocol. When the serum Ca levels became comparable between *B* and *C*, then the only difference in the major determinant of the whole/intact PTH ratio was the use of CH, and thus we could evaluate the pure effect of CH

Blood samples were obtained from the CH patients before the CH therapy was initiated (0 W), and at 4, 8, 12, 16, 24, and 48 weeks after the initiation of the CH therapy. Data obtained from the CH group were subjected to the analyses only at the time points when the serum calcium concentration was comparable to that in the control group (Fig. 1).

Intact PTH was detected by an electrochemiluminescence immunocomparable assay (Roche Diagnostics, Tokyo), and whole PTH was detected by an immunoradiometric assay (DS Pharma Biomedical Co., Osaka). For the control group, the blood sample collection was performed only once.

The data are expressed as the mean (SD). Comparisons between the control group and the CH group were performed with an unpaired *t* test. Comparisons within the CH groups were performed with a paired *t* test. A *p* value <.05 was considered significant.

**Fig. 2:**
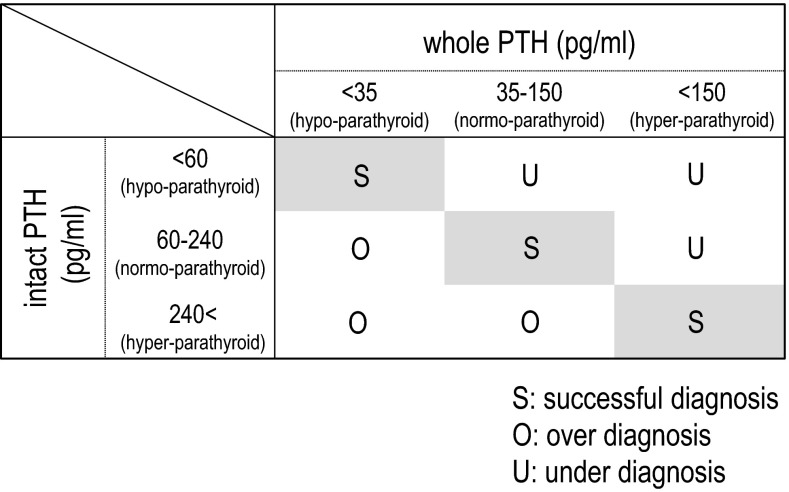
Analyzing principle used to assess the effect of the whole/intact PTH change in clinical practice. The JSDT guidelines set two standard PTH levels: intact PTH levels between 60 and 240 pg/mL and whole PTH levels between 35 and 150 pg/mL. If the relationship between these two assay results is always constant, the diagnoses made by these two standard levels would agree, and therefore the patients would be classified into successful diagnosis (*S*) in the majority of cases. However, if CH therapy modifies the relationship between these assay results, the numbers of cases classified into under-diagnosis (*U*) or over-diagnosis (*O*) may be increased, which becomes a potential cause of false diagnosis at bedside

In order to simulate the clinical influence of the alteration of the whole/intact PTH ratio by CH therapy, we performed an analysis as follows. Parathyroid function in the patients was diagnosed by their intact PTH levels according to the JSDT guidelines, namely, hypoparathyroid condition = intact PTH <60 pg/Ll, normoparathyroid condition = intact PTH levels between 60 and 240 pg/mL, and hyperparathyroid condition = intact PTH levels more than 240 pg/mL. Similarly, the patients were diagnosed by their whole PTH levels according to the JSDT guidelines, namely, hypoparathyroid condition = whole PTH levels <35 pg/mL, normoparathyroid condition = whole PTH levels between 35 and 150 pg/mL, and hyperparathyroid condition = whole PTH levels more than 150 pg/mL. When these diagnoses were in agreement with each other, the diagnosis was regarded as successful. Those cases with hypoparathyroid conditions measured by the intact PTH assay and normo- or hyperparathyroid conditions measured by the whole PTH assay, as well as those cases with hypo- or normoparathyroid conditions as shown by the intact PTH assay and hyperparathyroid condition shown by the whole PTH assay, were considered to have an underestimation of whole PTH by the conversion formula with intact PTH. The patients with normo- or hyperparathyroid conditions by intact PTH and hypoparathyroid condition by whole PTH, as well as those with hyperparathyroid condition by intact PTH and hypo- or normoparathyroid conditions by whole PTH, were considered to have an overestimation of whole PTH by the conversion formula with intact PTH (Fig. 2). The above analyses were performed in both the control and CH groups, and the ratios of successful diagnosis, under-diagnosis, and over-diagnosis were compared between them.

This study was designed in accordance with the Helsinki Declaration. All participants in both the control and CH groups received an adequate explanation of the study design, and written consent was obtained before the initiation of the study. This study protocol was approved by the Ethics Committee of Niigata University (#1028). The data presented in this manuscript were managed only by the authors or the cooperating personnel listed in the Acknowledgment section.

## Results

Sixty-seven patients were originally registered for the study as the CH group. Of these 67, CH treatment was discontinued due to over-suppression of parathyroid function in 11 patients, nausea in 4, transfer to another dialysis clinic in 4, and for other reasons in 2 patients. Parathyroid intervention therapy was indicated in 1 patient. Data were inappropriately collected from 1 patient. After excluding the above 23 patients, 44 patients were included in the final analysis. For the control group, 113 subjects were enrolled, and the data were appropriately collected from 112 patients who were included in the analysis. Table 1 shows the clinical back grounds of the CH group and control group patients. Before the CH treatment was initiated, the serum Ca levels were significantly higher in the CH group than those in the control group. The CH group also showed higher serum levels of intact PTH and whole PTH, and lower whole/intact PTH ratios.

**Table 1:** The clinical background of the patients in the CH group and control groups

	Control (*N* = 112)	CH (*N* = 44)	*p*
Sex	M70:F42	M28:F16	.896
Age	64.2 (12.7)	63.8 (12.4)	.835
Dialysis vintage (Y)	12.0 (5.5)	12.4 (7.9)	.697
Diabetes mellitus (%)	21.4	20.5	.894

**Fig. 3:**
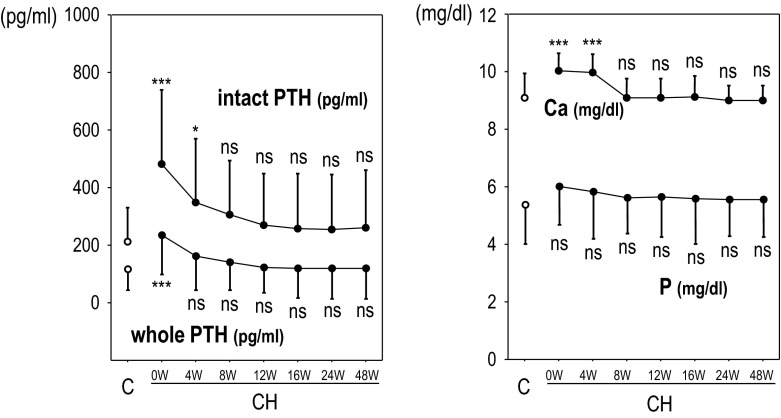
The changes in biochemical data during CH therapy. The serum levels of Ca, Pi, intact PTH, and whole PTH decreased along with the CH therapy. Although the CH group showed higher serum Ca levels before the initiation of the CH therapy, the levels became comparable with the control group at the 8th week and thereafter. *C* the control group, *CH* the cinacalcet hydrochloride group. *p < .05, ***p < .001 versus C

In the CH group, the dosages of active vitamin D agents, phosphate binders, and antihypertensive agents were generally unchanged in the CH group, but the dosage of CH was slightly increased along with the time course (Table 2). The serum levels of Ca, Pi, intact PTH, and whole PTH decreased along with the CH therapy. Although the CH group showed higher serum Ca levels before the initiation of CH therapy, the levels became comparable to the control group at the 8th week and thereafter (Fig. 3). Therefore, we compared the data obtained from the control group and the CH group at weeks 8, 12, 16, 24, and 48.

**Table 2:** The medication profile of the CH group patients and the control group

	Control	CH						
		0 W	4 W	8 W	12 W	16 W	24 W	48 W
CH (SD) (mg)	0	0	26.7 (6.4)	36.4 (14.7)	39.2 (15.6)	39.8 (16.5)	40.3 (17.2)	43.2 (16.5)
VDRA (%)	87.5	100	100	100	97.7	95.5	97.7	93.2
OPB (%)	75.0	86.4	86.4	86.4	84.1	84.1	81.8	86.4
AH (%)	74.1	77.3	77.3	77.3	77.3	75.0	77.3	79.5
ESA (%)	85.7	81.8	81.8	86.4	86.4	84.1	81.8	79.5

*CH* cinacalcet hydrochloride, *VDRA* vitamin D receptor activator, *AH* anti-hypertensive agents, *OPB* oral phosphate binders, *ESA* erythropoiesis stimulating agents

**Fig. 4:**
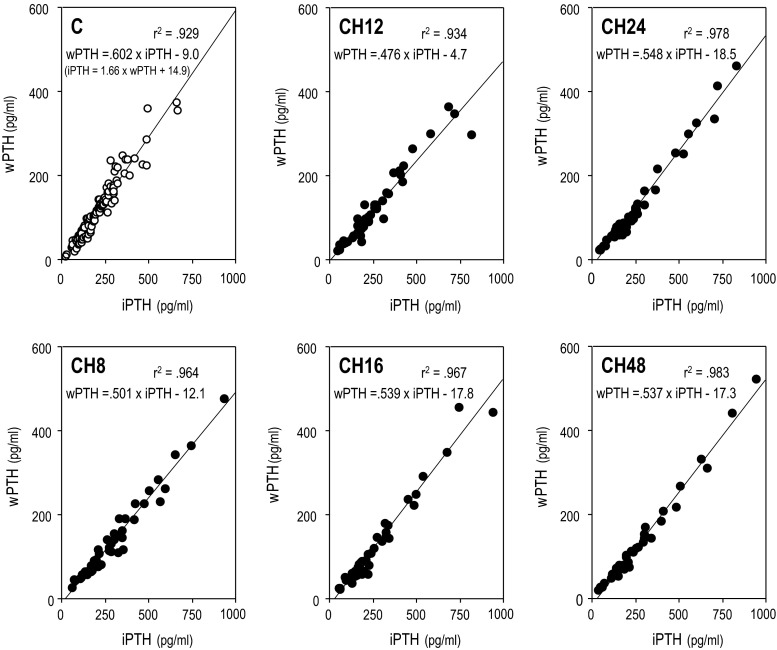
The relationship between intact PTH and whole PTH in the control and CH groups at weeks 8, 12, 16, 24, and 48. These values all showed tight correlations; however, the regression lines were steeper in the CH groups. *p < .05 versus the steepness in the control group

**Fig. 5:**
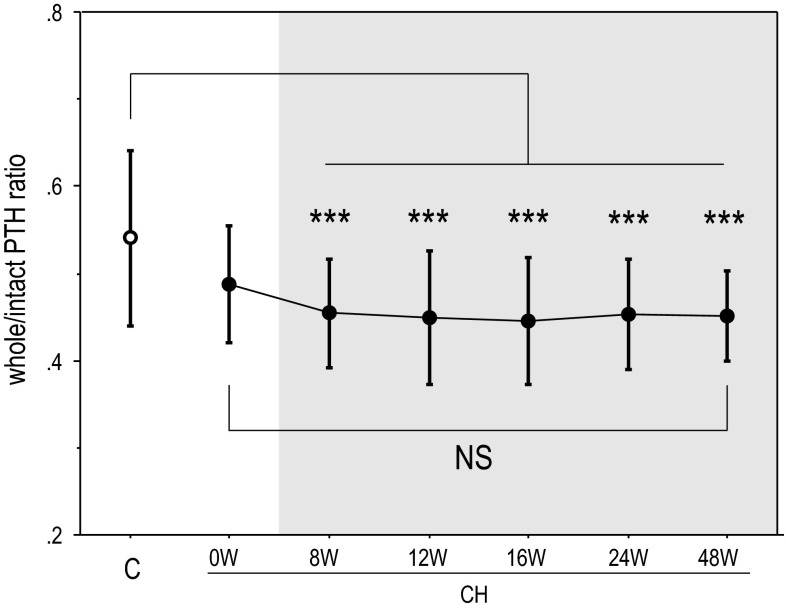
The comparison of the whole/intact PTH ratios. The CH at 8, 12, 16, 24, and 48 weeks showed significantly lower whole/intact PTH ratios than the control group. Although the serum Ca levels were different between the control and the CH groups at 0 W, the difference in the whole/intact PTH ratios did not reach significant level. The serum Ca levels were comparable between the control and CH groups at the 8th week and thereafter. Therefore, the difference in the whole/intact PTH ratios between these groups would be attributable solely to the CH therapy. On the other hand, the whole/intact PTH ratios were not significantly different between those before and during the CH therapy. **p < .01, ***p < .001 versus C

Figure 4 demonstrates the relationship between intact PTH and whole PTH in the control and CH groups at weeks 8, 12, 16, 24, and 48. Although these parameters were tightly correlated within both groups at all-time points, the regression lines were significantly less steep in the CH group. The CH group showed significantly lower whole/intact PTH ratios compared with the control group. However, the whole/intact PTH ratio was not significantly different between those before and during the CH therapy (Fig. 5). The results of the multiple regression analyses are demonstrated in Table 3. When we analyzed the control patients together with the CH group patients before the CH treatment, we identified the circulating levels of intact PTH and Ca as critical factors determining the circulating whole PTH levels. A comparable result was obtained when the control patients together with the CH group patients at the 48th week were analyzed with the same model. However, when CH treatment, the interaction term between intact PTH and CH (IT iPTH*CH), the interaction term between intact PTH and Ca (IT iPTH*Ca), and the interaction term between CH and Ca (IT CH*Ca) were added to the model as independent variables, only intact PTH, IT iPTH*CH, and IT iPTH*Ca were indicated as significant factors determining the circulating whole PTH levels.

**Table 3:** Results of the multiple regression analyses

	*T*	*p*	95 % CI
*(A)*			
iPTH	51.074	<.0001	.500 to .540
Ca	−4.374	<.0001	−14.954 to −5.647
Age	1.050	.296	−.155 to 506
Sex	.858	.392	−4.696 to 11.911
Vintage	.818	.415	.336 to 1.813
DM	.635	.526	−6.815 to 13.270
P	−.455	.650	−3.666 to 2.294
*(B)*			
iPTH	49.976	<.0001	.537 to .585
Ca	−2.068	.040	−9.132 to −.207
P	−1.464	.145	−4.548 to .677
Age	.842	.401	−.161 to 400
Vintage	−.455	.650	−.707 to 442
DM	.035	.972	−8.411 to 8.715
Sex	.002	.998	−7.080 to 7.094
*(C)*			
iPTH	8.862	<.0001	.857 to 1.349
IT iPTH*CH	−4.194	<.0001	−.122 to −.044
IT iPTH*Ca	−3.991	.0001	−.078 to −.026
Ca	1.849	.067	−.448 to 13.426
P	−1.775	.078	−4.022 to .216
Age	1.314	.191	−.075 to 372
DM	.618	.537	−4.748 to 9.071
CH	−.249	.803	−108.794 to 84.415
IT CH*Ca	.167	.867	−9.909 to 11.743
Sex	−.115	.909	−5.961 to 5.306
Vintage	.100	.920	−.037 to 041

*A* We analyzed the control patients together with the CH group patients before the CH treatment. Their age, sex, dialysis vintage, diabetes mellitus, circulating levels of intact PTH, Ca, and Pi were applied as the independent variables, and the whole PTH levels were set as the bound variable. As a result, intact PTH and Ca were identified as two significant factors determining the whole PTH levels

*B* The control patients together with the CH group patients at the 48th week were analyzed with the same model as that shown in A. Again, intact PTH and Ca were identified as two significant factors

*C* The CH treatment, the interaction term between intact PTH and CH (IT iPTH*CH), that between intact PTH and Ca (IT iPTH*Ca), and that between CH and Ca (IT CH*Ca) were added as independent variables to the model shown in B. Intact PTH, IT iPTH*CH, and IT iPTH*Ca were confirmed to be significant factors determining the whole PTH levels

*DM* comorbid condition of diabetes mellitus, *vintage* dialysis vintage

In the control group, the diagnosis of parathyroid condition obtained based on the intact PTH assay successfully accorded with that obtained based on the whole PTH assay in more than 90 % of the cases. In contrast, the rate of successful diagnosis declined to <80 % among those under CH therapy. All of the unsuccessful cases were overestimations of whole PTH by the conversion formula with intact PTH (Table 4).

**Table 4:** The effect of the CH therapy on the accordance of diagnoses made based on the intact PTH and whole PTH assay results

	Successful diagnosis (%)	Over diagnosis (%)	Under diagnosis
Cont	91.1	8.9	0
CH			
8 W	77.3	22.7**	0
16 W	79.5	20.5*	0
24 W	75.0	25.0**	0
48 W	77.3	22.7**	0

In the control group (C), the diagnosis of parathyroid condition by the intact PTH successfully accorded with that by whole PTH in more than 90 % of the cases. However, the rate of successful diagnosis fell to <80 % among those under the CH therapy (CH). All of the unsuccessful cases were overestimation of whole PTH by the conversion formula with intact PTH

* *p* < .05, ** *p* < .01 versus C

## Discussion

It is the extracellular Ca level that plays the central role in switching 1-84PTH/7-84PTH secretions in parathyroid cells [1, 7, 8], and CH may modify the mechanism through Ca-sensing receptor activation. Therefore, the switching of 1-84PTH/7-84PTH secretions in patients under CH therapy is controlled by two major factors: extracellular Ca and CH. The principal function of CH in parathyroid function is, strictly speaking, not the suppression of parathyroid cell function, but the alteration of the sigmoid curve between extracellular Ca stimuli and PTH secretion [15]. By considering this fact, we can explain why the previous research failed to demonstrate a change in the whole/intact PTH ratio by CH therapy [16]. CH suppressed 1-84PTH secretion and promoted 7-84PTH secretion. However, CH therapy also displayed hypocalcemic action [17], and the decrease in extracellular Ca levels promoted 1-84PTH secretion. Thus, Ca counteracted the 1-84PTH secretion against CH, and therefore, the effect of CH on the whole/intact PTH ratio became difficult to assess by comparing data before and during CH therapy in the same patients. In fact, just as in the previous study, the whole/intact PTH ratios before the CH therapy were comparable to those during the therapy in the present study as well (Fig. 3).

Thus, we established a group of control patients who did not need to be treated with CH, and we compared the data obtained from the CH group with that from the control group, instead of comparing the data obtained from the same patients before and during CH therapy. However, we have to admit that the selection criteria for the control patients were not clear enough for our scientific analyses, ant this is the largest limitation of this study.

To establish the control group, we selected patients with mild secondary hyperparathyroidism who did not need CH therapy. Since CH therapy decreases serum Ca levels, it is generally not chosen for relatively hypocalcemic patients. In the present study, therefore, relatively hypercalcemic patients were enrolled as the CH group. The difference in the whole/intact PTH ratios between the control group and CH group at 0 W could be explained by the difference in their serum Ca levels (Fig. 2).

Since the difference in serum Ca levels between the treatment and control groups disappeared at week 8, and thereafter, it became possible to compare the effects of CH at these time points. As a result, CH therapy was found to decrease the whole/intact PTH ratio (Fig. 2).

The intact PTH levels and whole PTH levels showed a tight correlation in the control group, as we expected, and the regression formula was as follows: intact PTH = 1.711 × whole PTH (Fig. 3). This formula is similar to the conversion formula that appeared in the original version of the JSDT guidelines: intact PTH = 1.7 × whole PTH [17]. The intact PTH levels and whole PTH levels also showed tight linear correlations in the CH group, but the steepness of the regression line increased according to the changes in the whole/intact PTH ratio. The results presented in Table 3C are expressed as follows:

$$ {\text{whole PTH}} = {\text{A1}} \times \left( {\text{intact PTH}} \right) + {\text{A2}} \times \left( {{\text{IT iPTH}}*{\text{CH}}} \right) + {\text{A3}} \times \left( {{\text{IT iPTH}}*{\text{Ca}}} \right) = ({\text{A1}} + {\text{A2}} \times {\text{CH}} + {\text{A3}} \times {\text{Ca}}) \times {\text{int PTH}}, $$

where A1, A2, and A3 indicate fixed numbers, respectively. This formula is converted as: the whole PTH/intact PTH ratio = A1 + A2 × CH + A3 × Ca. Therefore, CH was indicated as a potential bias besides Ca in the estimation of the whole PTH levels from the intact PTH levels.

Table 4 demonstrates the potential effect of the above changes in clinical practice. The diagnosis by intact PTH accorded with that by whole PTH in over 90 % of the patients in the control group. This result supported the conventional concept that parathyroid function could be assessed by either the intact PTH or the whole PTH. The JSDT guidelines set the standard parathyroid function range of both intact PTH and whole PTH levels based on the premise that these two diagnoses always agree. However, the evidence adopted in these clinical practice guidelines was all obtained before CH became available in clinical practice [14, 18]. Therefore, the relationship between intact PTH and whole PTH described by the JSDT guidelines is that between these levels under a condition in which CH is not administered, namely, the relationship found in the control group in the present study.

On the other hand, the agreement of these two diagnoses was decreased to <80 % in the CH group. All the disagreement cases overestimated the whole PTH levels by intact PTH levels (Table 4). This result was caused by the alteration of the relationship between intact PTH and whole PTH brought about by the CH therapy; in other words, the standard PTH levels expressed with intact PTH and those expressed with whole PTH indicate differing parathyroid functions under this condition. Since these patients were being treated with CH because of hyperparathyroidism, the impact of the potential unreliability of PTH assessment is great. If intact PTH levels indicate the true parathyroid function, the parathyroid suppression therapy would become under-treatment in patients whose parathyroid functions are monitored by whole PTH levels. If whole PTH indicates the true parathyroid function, CH therapy may tend to become over-treatment in those patients for whom intact PTH is used as a monitoring tool.

At present, it is uncertain whether intact PTH or whole PTH more accurately indicates true parathyroid function. However, the conventional concept that we can use either intact PTH or whole PTH for parathyroid monitoring cannot be applied under these conditions. Therefore, the time has come to choose the better PTH assay for standard use. In theory, the whole PTH assay seems to show stable results even under these conditions. Moreover, the intact PTH assays show greater interassay heterogeneities than the whole PTH assays [19, 20]. However, further clinical investigations will be needed to select the better choice for bedside use.

In conclusion, the whole/intact PTH ratio in the dialysis patients under CH therapy was significantly lower than that in the control dialysis patients with comparable serum Ca levels. For patients under CH therapy, we must consider the most appropriate assay to assess parathyroid function, and we should not blindly apply the present clinical practice guidelines.
